# Changes in Serum Concentrations of Fibroblast Growth Factor 23 and Soluble Klotho in Hemodialysis Patients after Total Parathyroidectomy

**DOI:** 10.1155/2016/6453803

**Published:** 2016-11-24

**Authors:** Shang-Chih Liao, Sin-Hua Moi, Fong-Fu Chou, Cheng-Hong Yang, Jin-Bor Chen

**Affiliations:** ^1^Division of Nephrology, Kaohsiung Municipal Feng-Shan Hospital, Kaohsiung, Taiwan; ^2^Department of Electronic Engineering, National Kaohsiung University of Applied Sciences, Kaohsiung, Taiwan; ^3^Department of Surgery, Kaohsiung Chang Gung Memorial Hospital and Chang Gung University College of Medicine, Kaohsiung, Taiwan; ^4^Division of Nephrology, Department of Internal Medicine, Kaohsiung Chang Gung Memorial Hospital and Chang Gung University College of Medicine, Kaohsiung, Taiwan

## Abstract

*Background*. We examined the changes in circulating fibroblast growth factor 23 (FGF23) and Klotho concentrations in hemodialysis patients after parathyroidectomy (PTX).* Methods*. We enrolled a cohort of hemodialysis patients who received PTX. Postoperatively, patients received calcium supplements and/or vitamin D analogue (calcitriol) to maintain serum calcium within 7.0–8.0 mg/dL. Information on clinical parameters including bone-mineral metabolic variables was collected pre-PTX and on days 5 and 90 after PTX. Concomitantly, serum full-length FGF23 and *α*-Klotho levels were measured. The relationship between FGF23 and clinical parameters was analyzed by single linear regression.* Results*. Forty-six participants (33 women; 13 men) were enrolled in the study. Their mean age was 56.49 years. Serum FGF23 and *α*-Klotho concentrations were elevated on days 5 and 90 after PTX compared to baseline (*p* > 0.05). Serum FGF23 concentrations negatively correlated with serum calcium concentrations pre-PTX (Beta −0.31; *R* ^2^ 0.0949; *p* = 0.040), day 5 post-PTX (Beta −0.31; *R* ^2^ 0.0982; *p* = 0.036), and day 90 post-PTX (Beta −0.39; *R* ^2^ 0.1528; *p* = 0.008).* Conclusions*. There was no change in circulating FGF23 and Klotho concentrations after PTX in hemodialysis patients given postoperative calcium supplements and/or vitamin D analogue. Serum FGF23 concentrations pre-PTX and at days 5 and 90 after PTX were inversely related to serum calcium concentrations.

## 1. Introduction

Secondary hyperparathyroidism (SHPT) is a common complication of chronic kidney disease (CKD) and occurs as a consequence of calcium, phosphate, and vitamin D homeostasis. Recently, fibroblast growth factor 23 (FGF23) has been identified as a protein which plays a crucial role in phosphate regulation. FGF23 is primarily secreted by osteocytes and is involved in controlling the metabolism of phosphate, parathyroid hormone (PTH), and 1,25 dihydroxyvitamin D (1,25(OH)_2_D_3_) [1, 2]. In the early stage of CKD, elevated FGF23 levels increase fractional phosphate excretion and subsequently reduce serum phosphate and 1*α*-hydroxylase levels. These changes in turn reduce 1,25(OH)_2_D_3_ formation and increase PTH secretion [3–5]. In the late stage of CKD, FGF23 cannot regulate phosphate homeostasis and it adversely affects outcome in CKD by contributing to disease progression, left ventricular hypertrophy, and increased mortality [5–8].

Klotho is an antiaging gene, which encodes a transmembrane protein that forms a complex with multiple growth factor receptors [9]. FGF23 exerts its effects by forming a complex with Klotho [10]. Klotho also acts as a humoral factor when it is cleaved and released into the circulation. Previous studies have demonstrated that the circulating soluble form of Klotho can enhance the excretion of phosphate in the proximal nephron and promote calcium reabsorption in the distal nephron [11, 12]. The concentrations of circulating FGF23 and Klotho were found to progressively decline after parathyroidectomy (PTX). However, circulating Klotho levels only decreased transiently before increasing progressively thereafter [13].

The literature on serial changes in circulating FGF23 and Klotho concentrations after PTX in hemodialysis (HD) patients is scarce, and thus we conducted a study to monitor these values in a cohort of HD patients after PTX. We aimed to examine the changes in circulating FGF23 and Klotho concentrations until 90 days after PTX. We also evaluated the relationships between clinical parameters and circulating concentrations of FGF23 and Klotho after PTX.

## 2. Materials and Methods

### 2.1. Patients

We enrolled HD patients who received PTX at Kaohsiung Chang Gung Memorial Hospital in Taiwan. The patients were tracked from January 1, 2014, until December 31, 2016. The inclusion criteria were (1) patients receiving regular HD for at least 3 months and (2) follow-up for at least for 3 months after PTX. The exclusion criteria were (1) patient age greater than 90 years old; (2) patients transferred to other medical facilities; and (3) patients whose information was incomplete and/or those who were lost to follow-up after PTX. The indications for PTX included uncontrolled pruritus, generalized bone pain, resistance to medical treatment, and high intact PTH (iPTH) levels (>1000 pg/mL). The PTX procedure included a total parathyroidectomy and autotransplantation of 140 mg of hyperplastic tissue of the parathyroid gland into subcutaneous forearm tissue. All patients received calcium supplements and/or vitamin D analogue (calcitriol) on day 1 after PTX to maintain serum calcium concentrations around 7.0–8.0 mg/dL.

### 2.2. Laboratory Measurements

Routine hematological and biochemical investigations were performed one day prior to PTX (baseline). Serum albumin, calcium (Ca), phosphate (P), alkaline phosphatase (ALP), and bone-alkaline phosphatase (B-ALP) concentrations were measured again at 5 days and 90 days after PTX. All blood samples were measured using commercial kits and an autoanalyzer (Hitachi 7600-210, Hitachi Ltd., Tokyo, Japan). Albumin levels were measured using the bromocresol green method. ALP was measured by colorimetric assay as the hydrolysis of* p*-nitrophenyl phosphate according to instructions from the supplier (Roche Diagnostic Indianapolis, USA). B-ALP was measured by chemiluminescence immunoassay (DiaSorin LIAISON® BAP OSTASE®, DiaSorin Inc., USA). Serum full-length FGF23 concentration was measured using a commercially available enzyme-linked immunosorbent assay (ELISA) kit (SunLong Biotech, China). Serum Klotho concentration was measured using human soluble *α*-Klotho assay kit (Immuno-Biological Laboratories, Japan). Serum 25-hydroxyvitamin D concentration was measured using human soluble 25-hydroxyvitamin D assay kit (DiaSorin, USA).

The protocol for the present study was approved by the Committee on Human Research at Kaohsiung Chang Gung Memorial Hospital (102-2891B) and funded by the Chang Gung Memorial Hospital Grant (CORPG8C1202). The study was conducted in accordance with the Declaration of Helsinki. All study participants provided written informed consent.

### 2.3. Statistics

The baseline characteristics were summarized as total number, percentage, and mean, SD. Changes in FGF23 and *α*-Klotho before PTX and after PTX (at 5 and 90 days) were evaluated by independent one-way repeated ANOVA. The FGF23 and *α*-Klotho measurements before PTX and after PTX (day 5 and day 90) were visually summarized using a boxplot graph. We evaluated the relationship between FGF23 and other parameters using single linear regression. A *p* value < 0.05 was considered statistically significant. All statistical analyses were performed by STATA (version 11.1).

## 3. Results

### 3.1. Participant Demographic Characteristics

A total of 46 participants were enrolled in the final study. The mean age of the study population was 56.49 years, and the ratio of women : men was 33 : 13. The majority of patients (*n* = 40/46) had comorbid hypertension. Laboratory parameters at the baseline showed markedly elevated levels of ALP, B-ALP, and iPTH (Table 1).

**Figure 1:**
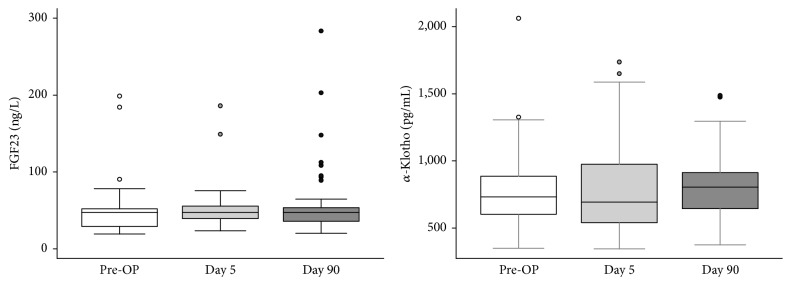
The serum concentrations of FGF23 and *α*-Klotho in the study period. The boxes represent the medium values and the vertical lines the maximum and minimum values in the participants measured.

Compared to baseline, there was no significant increase in the serum concentrations of FGF23 and *α*-Klotho at 90 days after PTX (Figure 1) (the detailed data sheet was shown in the Supplementary Table 1 in Supplementary Material available online at http://dx.doi.org/10.1155/2016/6453803).

### 3.2. Correlation between FGF23 Concentrations and Mineral Bone Metabolism Parameters

We performed single linear regression analysis to investigate the relationship between serial changes in FGF23 concentrations (baseline and 5 and 90 days post-PTX) and mineral bone metabolism parameters. There was a significant negative correlation between serum FGF23 concentrations and serum calcium concentrations at baseline (Beta −0.31; *R* ^2^ 0.0949; *p* = 0.040), at 5 days post-PTX (Beta −0.31; *R* ^2^ 0.0982; *p* = 0.036), and at 90 days post-PTX (Beta −0.39; *R* ^2^ 0.1528; *p* = 0.008) (Table 2). We assumed a closer approximation to a standard distribution curve when FGF23 concentrations were transformed to logarithms. The results showed increased correlation between log⁡FGF23 concentrations and serum creatinine concentrations from baseline to 90-day PTX (no statistical significance). Serum log⁡FGF23 concentrations were negatively correlated with serum calcium concentrations from baseline to 90 days post-PTX (only significant at 90 days post-PTX, Beta −0.32; *R* ^2^ 0.1002; *p* = 0.034). Moreover, there were no significant correlations between either FGF23 or log⁡FGF23 concentrations and mineral bone metabolism markers [P, calcium-phosphate product (CaxP), intact PTH, ALP, B-ALP, 25(OH)_2_D_3_] from baseline to 90-day PTX (Table 2).

### 3.3. Relationship between FGF23 and *α*-Klotho Concentrations

**Figure 2:**
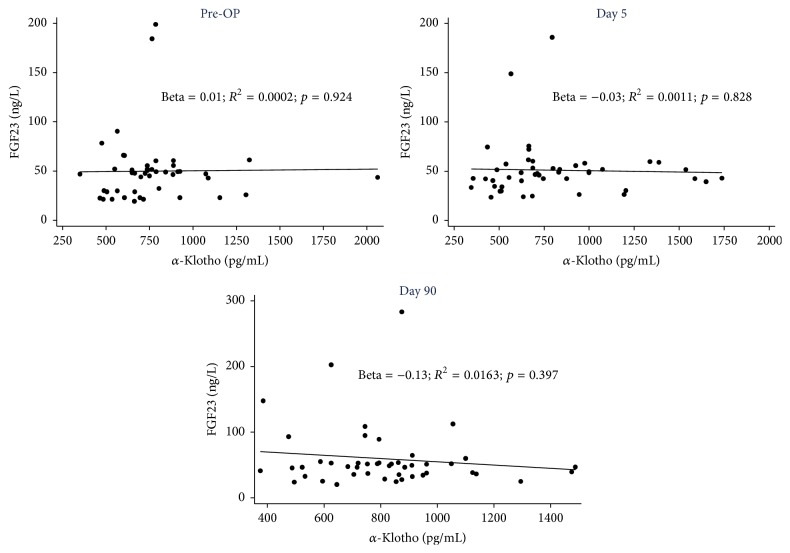
The correlation between serum FGF23 and *α*-Klotho concentrations in the study period.

FGF23 concentrations and *α*-Klotho concentrations were not significantly correlated at any time point (baseline and 5 and 90 days post-PTX) (Figure 2).

## 4. Discussion

In the present study, we examined the serial changes in serum FGF23 and *α*-Klotho concentrations in HD patients after PTX. There were no significant changes in the concentrations of FGF23 or *α*-Klotho at baseline, 5 days post-PTX, or 90 days post-PTX. In addition, we did not find a significant correlation between FGF23 and *α*-Klotho concentrations during the study period. These results differ from previous studies, which showed a progressive decrease in FGF23 concentrations from pre-PTX to 10 days after PTX [13, 14]. In another study, FGF23 concentrations at days 1 and 3 correlated with serum phosphorus and calcium-phosphorus levels [14]. One report from Takahashi et al. [13] showed a progressive decline in FGF23 concentrations together with significant reductions in serum Ca, P, and iPTH concentrations from pre-PTX to 30 days after PTX and stable FGF23 concentrations from 30 to 90 days after PTX. This is in contrast to the results of another study, which examined changes in FGF23 levels after PTX for primary hyperparathyroidism and demonstrated no change between day 1 and 6 weeks after PTX. Meanwhile, serum FGF23 concentrations did not correlate with serum P, Ca, iPTH, 1*α*,25(OH)_2_D, or B-ALP concentrations in the pre-PTX and post-PTX state [15]. The present study measured serum FGF23 concentrations at three time points: pre-PTX and 5 days and 90 days after PTX. Our patients received calcium supplements and vitamin D analogue (calcitriol) after PTX to keep serum calcium concentrations in the range of 7.0–8.0 mg/dL. It is worth noting that certain diets and medications can also modify FGF23 concentrations including a vegetarian diet, phosphate restriction or phosphate binders, vitamin D analogues, parathyroidectomy, cinacalcet, and kidney transplantation [16]. In parathyroidectomized rats, calcitriol significantly increased FGF23 levels [17]. We speculated that calcitriol use in our patients likely contributed to the increased FGF23 concentrations observed after PTX. In a study of healthy volunteers, high doses of inorganic phosphate failed to trigger a rise in serum FGF23 concentrations despite significant increases in renal phosphate clearance [18]. These data imply that serum FGF23 concentrations are susceptible to regulation by long-term serum phosphate levels but not to acute changes in serum phosphate concentration. Since our patients reached a well-controlled phosphate status after PTX, it is possible that they were resistant to rises in serum FGF23 concentrations.

Klotho was originally identified as an aging suppressor gene [9]. In CKD, Klotho may ameliorate vascular calcification by enhancing phosphaturia, and it has also been shown to directly inhibit phosphate uptake by vascular smooth muscle and preserve glomerular filtration [19]. *α*-Klotho functions as a coreceptor for FGF23 [10, 20, 21] and is secreted into the blood, urine, and cerebrospinal fluid [11, 22]. The *α*-Klotho gene product is expressed in a membrane-bound form (mKL) in association with FGF23 and FGF receptors (FGFRs) and signals through the MAPK cascade [10, 20]. Two soluble species have been reported: an alternatively spliced secreted form (sKL) and an endoproteolytic cleavage product of mKL (cKL) [23]. Only the cKL protein was detectable in human and rodent plasma and cerebrospinal fluid [22]. cKL stimulates FGF23 production* in vivo* and results in elevated FGF23 levels [24]. Takahashi et al. [13] reported a 13% decrease from baseline in serum soluble Klotho concentrations on the day after PTX and a subsequent increase to peak levels of 34% above baseline at 90 days post-PTX [13]. The present study showed a nonsignificant increase in serum *α*-Klotho concentrations after PTX on days 5 and 90 (Supplementary Table 1). This is in keeping with the findings reported by Takahashi et al. [13].

We found a negative correlation between FGF23 concentrations and serum calcium concentrations at pre-PTX and days 5 and 90 after PTX. However, there was no significant correlation between FGF23 concentrations and serum P, iPTH, or 25(OH) D_3_ concentrations. This is in contrast to the report by Sato et al. [14], which demonstrated a correlation between FGF23 concentrations on days 1 and 3 after PTX and serum phosphate and calcium-phosphate product levels [14]. Of note, serum calcium concentrations decreased to less than 8 mg/dL on day 3 after PTX and calcium supplements were not administrated to any patient before day 2 in the aforementioned study [14]. In contrast, our patients received calcium supplements and calcitriol one day after PTX to maintain serum calcium concentrations above 7.0 mg/dL. It is possible that this discrepancy in study protocol contributed to the different results obtained between the two studies.

The present study has some limitations. Firstly, our patients received calcium supplements and/or vitamin D analogue one day after PTX. These interventions may alter calcium-phosphate homeostasis in dialysis patients and further complicate the complex regulation of FGF23 and Klotho post-PTX. Therefore, it is not yet possible to accurately outline the mechanism whereby PTH regulates FGF23 and Klotho production after PTX in dialysis patients. Secondly, a study in wild-type mice placed on an iron-deficient diet showed an increase in* Fgf23* mRNA expression in bone, as well as increased circulating levels of C-terminal FGF23 fragments in the absence of any change in intact FGF23 levels [25]. Since the present study measured serum full-length FGF23 concentrations, we cannot be certain of the contribution of circulating FGF23 fragments to the values obtained in the assay. A similar problem was encountered in the measurement of circulating Klotho concentrations. It would be useful to obtain a response curve of circulating Klotho levels after PTX in dialysis patients using separated component measurements for Klotho. Furthermore, the present study did not measure circulating cKL concentrations, which may play a role in stimulating FGF23 production [24]. Furthermore, this clinical study cannot propose a mechanism to explain how FGF23 regulates bone-mineral metabolism after PTX. Finally, the present study enrolled a relatively small number of participants. A well-designed randomized controlled study enrolling more participants is needed to further validate the results of the current study. Despite the limitations stated above, the present study provides a clinically relevant analysis of changes in FGF23 and Klotho levels after PTX in dialysis patients. Future studies aimed at following up these changes over a longer period of time and delineating the interactions between calcium supplements/vitamin D analogue and FGF23/Klotho are warranted.

## 5. Conclusions

The present study demonstrated no change in circulating FGF23 and Klotho concentrations after PTX in HD patients receiving calcium supplement and/or vitamin D analogues postoperatively. There was a negative correlation between serum FGF23 and calcium concentrations at pre-PTX and on days 5 and 90 after PTX.

## Supplementary Material

FGF23 and α-Klotho concentrations were not significantly changed after PTX. Concentrations of serum Ca, P, iPTH and CaxP values demonstrated significantly decreased after PTX.

## Figures and Tables

**Table 1:** Baseline characteristics in study subjects (*n* = 46).

Variables	*n*	%
Age (yrs) (mean, SD)	56.49	9.95
Sex		
Female	33	71.74
Male	13	28.26
HD vintage (yrs) (mean, SD)	6.14	4.31
Vitamine D use	8	16.33
Calcium salt use	8	16.33
Comorbidity		
Diabetes mellitus	6	13.04
Hypertension	40	86.96
Coronary	2	4.35
SBP (mean, SD)	139.10	26.46
DBP (mean, SD)	78.10	14.04
Lab parameters (mean, SD)		
Hb (g/dL)	11.13	1.73
Hct (%)	33.97	5.18
Albumin (g/dL)	4.82	4.59
BUN	41.39	20.99
Cr	9.21	10.58
Ca (mg/dL)	9.82	1.33
P (mg/dL)	5.51	1.91
CaxP (mg^2^/dL^2^)	54.40	20.52
ALP (U/L)	229.94	197.18
Bone-ALP (*μ*g/L)	65.00	39.43
Al (*μ*g/dL)	2.26	1.14
Ferritin (ng/mL)	363.84	234.53
iPTH (pg/ml)	1391.22	746.68
25(OH)D_3_ (ng/mL)	14.54	7.48

HD, hemodialysis; SBP, systolic blood pressure; DBP, diastolic blood pressure; Hb, hemoglobin; Hct, hematocrit; BUN, blood urea nitrogen; Cr, creatinine; Ca, calcium; P, phosphate; CaxP, calcium-phosphate product; ALP, alkaline phosphatase, Al, aluminum; iPTH, intact parathyroid hormone.

Reference range: Hb, 12.0–17.5 g/dL; Hct, 36–53%; Albumin, 3.5–5.0 g/dL; BUN, 6.0–21 mg/dL; Cr, 0.44–1.27 mg/dL; Ca, 7.9–9.9 mg/dL; P, 2.4–4.7 mg/dL; ALP, 40–140 U/L; Bone-ALP, 5.5–22.9 *μ*g/L; Al, <2.0 *μ*g/dL; Ferritin, 10–322 ng/mL; iPTH, 14–72 pg/ml; 25(OH)D_3_, 9.7–41.7 ng/mL.

**Table 2:** Relationships between FGF-23 and clinical parameters.

Variable	Baseline			Day 5			3 months		
	Beta	*R* ^2^	*p* value	Beta	*R* ^2^	*p*-value	Beta	*R* ^2^	*p* value
FGF-23									
Cr	−0.13	0.0161	0.402	−0.13	0.0162	0.400	0.15	0.0215	0.331
Ca (mg/dL)	−0.31	0.0949	0.040	−0.31	0.0982	0.036	−0.39	0.1528	0.008
P (mg/dL)	−0.01	0.0001	0.949	−0.06	0.0038	0.687	−0.02	0.0006	0.874
iPTH (pg/ml)	−0.10	0.0098	0.514	−0.11	0.0121	0.468	−0.09	0.0075	0.567
25(OH)D_3_ (ng/mL)	0.09	0.0087	0.538	0.07	0.0049	0.645	0.22	0.0488	0.140
ALP (U/L)	0.15	0.0217	0.414	0.27	0.0714	0.133	0.24	0.0589	0.174
Bone-ALP (*μ*g/L)	0.23	0.0521	0.413	0.31	0.0990	0.253	0.07	0.0053	0.797
CaxP (mg^2^/dL^2^)	−0.09	0.008	0.556	−0.14	0.018	0.375	−0.125	0.0156	0.413
log⁡FGF-23									
Cr	−0.19	0.0355	0.210	−0.18	0.0314	0.238	0.20	0.0415	0.174
Ca (mg/dL)	−0.21	0.0440	0.167	−0.22	0.0487	0.145	−0.32	0.1002	0.034
P (mg/dL)	0.09	0.0080	0.559	−0.01	0.0001	0.949	0.11	0.0129	0.458
iPTH (pg/ml)	−0.04	0.0018	0.778	−0.10	0.0097	0.515	−0.07	0.0044	0.661
25(OH)D_3_ (ng/ml)	−0.08	0.0063	0.600	−0.08	0.0058	0.615	0.12	0.0153	0.413
ALP (U/L)	0.12	0.0150	0.497	0.25	0.0626	0.160	0.18	0.0339	0.305
Bone-ALP (*μ*g/L)	0.15	0.0216	0.601	0.25	0.0642	0.362	−0.08	0.0059	0.786
CaxP (mg^2^/dL^2^)	0.03	0.0007	0.859	−0.06	0.0041	0.677	0.02	0.0003	0.903
