# Supplementary material for: Changes in Serum Concentrations of Fibroblast Growth Factor 23 and Soluble Klotho in Hemodialysis Patients after Total Parathyroidectomy
Source: Biomed Res Int. 2016 Nov 24;2016:6453803. doi: 10.1155/2016/6453803 (PMC5143687; doi:10.1155/2016/6453803)
Supplement: Supplementary file 1 — FGF23 and α-Klotho concentrations were not significantly changed after PTX. Concentrations of serum Ca, P, iPTH and CaxP values demonstrated significantly decreased after PTX. [file 6453803.f1.doc]

Supplementary Table 1: Serum concentrations of FGF23 and α-Klotho at 90 days after PTX

| Variables |  | Pre-OP | |  | 5-day | |  | 90-day | |  | *p*-value |
| --- | --- | --- | --- | --- | --- | --- | --- | --- | --- | --- | --- |
|  |  | Mean | SD |  | Mean | SD |  | Mean | SD |  |  |
| FGF23 (ng/L) |  | 49.86 | 34.58 |  | 51.11 | 28.43 |  | 59.45 | 47.81 |  | 0.715 |
| α-Klotho (pg/mL) |  | 768.58 | 288.04 |  | 807.45 | 356.11 |  | 817.17 | 247.56 |  | 0.42 |
| Ca (mg/dL) |  | 9.89 | 1.22 |  | 7.44 | 0.97 |  | 8.38 | 1.46 |  | <0.001 |
| P (mg/dL) |  | 5.48 | 1.89 |  | 4.23 | 1.43 |  | 3.31 | 1.41 |  | <0.001 |
| CaXP (mg^2^/dL^2^) |  | 54.45 | 20.04 |  | 31.47 | 11.75 |  | 27.67 | 13.36 |  | <0.001 |
| iPTH (pg/ml), median (interquartile range) |  | 1176.50 | (931-1587) |  | 9.39 | (4.55-18.2) |  | 76.85 | (19.2-347) |  | <0.001 |

*P*-value was estimated from one-way repeated ANOVA.
